# A Signature of N^6^-methyladenosine Regulator-Related Genes Predicts Prognoses and Immune Responses for Head and Neck Squamous Cell Carcinoma

**DOI:** 10.3389/fimmu.2022.809872

**Published:** 2022-02-03

**Authors:** Junjun Chen, Tianzhu Lu, Fangyan Zhong, Qiaoli Lv, Min Fang, Ziwei Tu, Yulong Ji, Jingao Li, Xiaochang Gong

**Affiliations:** ^1^ National Health Commission (NHC), Key Laboratory of Personalized Diagnosis and Treatment of Nasopharyngeal Carcinoma, Jiangxi Cancer Hospital of Nanchang University, Nanchang, China; ^2^ Department of Science and Education, Jiangxi Key Laboratory of Translational Cancer Research, Jiangxi Cancer Hospital of Nanchang University, Nanchang, China; ^3^ Department of Radiation Oncology, Jiangxi Cancer Hospital of Nanchang University, Nanchang, China

**Keywords:** signature, m6A regulators related genes, prognosis, immune responses, head and neck squamous cell carcinoma

## Abstract

This study aimed to construct a signature of N^6^-methyladenosine (m6A) regulator-related genes that could be used for the prognosis of head and neck squamous cell carcinoma (HNSCC) and to clarify the molecular and immune characteristics and benefits of immune checkpoint inhibitor (ICI) therapy using the prognostic signature to define the subgroups of HNSCC. This study showed that eighteen m6A regulators were abnormally expressed in the Cancer Genome Atlas (TCGA) HNSCC tissues compared with those in normal tissues. We constructed a signature of 12 m6A regulator-related genes using the Cox risk model, combined with the least absolute shrinkage and selection operator (Lasso) variable screening algorithm. Based on the median of the signature risk score, the patients were divided into high- and low-risk groups. The Kaplan–Meier survival analyses showed that patients with high-risk scores demonstrated poorer overall survival (OS) than those with low-risk scores based on TCGA-HNSCC data (*p <*0.001). The OS of high-risk patients was significantly worse than that of low-risk patients in the GSE65858 (*p <*0.001) and International Cancer Genome Consortium (ICGC) oral cancer cohorts (*p* = 0.0089). Furthermore, immune infiltration analyses showed that 8 types of immune cell infiltration showed highly significant differences between the two risk groups (*p <*0.001). In the Imvigor210CoreBiologies dataset of patients who received ICIs, the objective response rate (ORR) of the low-risk group (32%) was significantly higher than that of the high-risk group (13%). Additionally, patients in the high-risk group presented with a more significant adverse OS than that of the low-risk group (*p* = 0.00032). GSE78220 also showed that the ORR of the low-risk group (64%) was higher than that of the high-risk group (43%) and the OS of low-risk patients was better than that of high-risk patients (*p* = 0.0064). The constructed prognostic signature, based on m6A regulator-related genes, could be used to effectively distinguish between prognoses for HNSCC patients. The prognostic signature was found to be related to the immune cell infiltration of HNSCC; it might help predict the responses and prognoses of ICIs during treatment.

## Introduction

Head and neck squamous cell carcinoma (HNSCC) is a commonly occurring malignancy reported in humans worldwide (1, 2). Presently, most patients are diagnosed in the later stages, which often leads to a high risk of recurrence and metastatic disease development; the 5-year survival rates remain at 40%–50% (3, 4). The current standard management for HNSCC includes the assessment of patients’ prognoses based on the size, location, and invasion of their tumors using the Tumor, Nodes, and Metastases (TNM) classification system; strategies are formulated on this basis (5). However, patients within the same TNM stage continue to demonstrate different responses to treatment (6). Therefore, it is imperative to discover stable and reliable molecular signatures to evaluate the prognoses of patients and to propose more effective treatments.

N^6^-methyladenosine (m6A) is the most common post-transcriptional RNA modification documented in eukaryotic cells; these modifications mostly occur near mRNA stop codons in the 5′- and 3′-untranslated regions (UTRs), as well as in internal long exons (7, 8). The m6A methylation level is coordinated by the action of methyltransferases (“writers”) such as the enzymes methyltransferase (METTL)3, METTL14, Wilms tumor 1-associated protein (WTAP), and VIRMA; by the action of demethylases (“erasers”) such as fat mass- and obesity-associated protein (FTO) and α-ketoglutarate-dependent dioxygenase AlkB homolog 5 (ALKBH5); and by the action of binding protein “readers” such as YTH domain-containing 1 (YTHDC1), YTH domain-containing 2 (YTHDC2), YTH N6-methyl-adenosine RNA binding protein 1 (YTHDF1), YTH N6-methyladenosine RNA binding protein 2 (YTHDF2), and heterogeneous nuclear ribonucleoprotein C (HNRNPC) (9–11). M6A methylation plays an essential role in many physiological and pathological processes, including immune response generation, microRNA editing, and the progression of various cancers (12–14).

The epigenetic modification of m6A methylation results in the regulation of tumor progression by affecting the expression of oncogenes or suppressor genes. For example, METTL3 expression is significantly upregulated in bladder cancer and is closely related to poor prognoses in patients (15). Moreover, many studies have shown that m6A methylation is closely related to anti-tumor immune response generation. Han et al. have demonstrated that YTHDF1 is an essential mediator of tumor immune evasion; their findings suggest that it may be a potential target for improving the efficacy of immunotherapy (16). Previously, Yi found that the signature based on m6A regulators can distinguish the prognosis of HNSCC patients (17). Simultaneously, the study found that some m6A regulators were correlated with the expression of PD-L1 in HNSCC. This means that m6A methylation played a vital role in the tumor immune microenvironment of HNSCC. However, due to the limited number of m6A regulators, the accuracy of this model for evaluating the prognosis of HNSCC is not ideal. The m6A regulators can extensively regulate the expression of downstream genes through m6A methylation. Therefore, constructing a prognostic risk model based on m6A regulator-related genes may better evaluate prognosis and immunotherapy response.

Here, first, m6A regulators related to genes were identified using TCGA database. Next, 12 genes were screened through univariate Cox analysis and the least absolute shrinkage and selection operator (Lasso) regression. A risk prognosis signature was then established through multivariate Cox regression analysis. Finally, the accuracy of the signature was verified in both the training and validation sets, and the relationship between the prognostic signature and immunotherapy responses was explored. In conclusion, it is anticipated that this study will provide insights for the development of a stable tool regarding prognosis and immunotherapy predictions that will be valuable for HNSCC patients.

## Materials and Methods

### Data Availability and Analysis

The data on genome copy number variation, gene mutation, gene expression, and methylation levels were downloaded from the TCGA-HNSCC database (https://xenabrowser.net/datapages/). For transcriptome data, 546 TCGA-HNSCC samples were used, including 500 tumor tissue samples, 44 normal tissue samples, and two metastatic cancer tissue samples. After removing the two metastatic cancer samples, 544 samples were used for gene expression correlation analysis. The clinical information of HNSCC patients in TCGA is provided in **Table 1**. All 522 copy number variation samples from the Gistic2 database were used for correlation analysis. For acquiring TCGA-HNSCC methylation data, the methylation beta value was directly used for correlation analysis; it was downloaded from TCGA-HNSCC database UCSC Xena (https://xenabrowser.net/datapages/). Additionally, the immune infiltration data of TCGA-HNSCC samples were obtained from http://timer.comp-genomics.org. M6A methylation modification sites were predicted according to the data and software provided by http://www.cuilab.cn/sramp. GSE65858 and GSE78220 datasets were acquired from Gene Expression Omnibus (GEO) and International Cancer Genome Consortium (ICGC) oral cancer from India (ORCA-IN) data. The immune response data were obtained using the R package “IMvigor210CoreBiologies” (v.1.0.0) (18). Data information used in this study is shown in **Supplementary Table 1**.

**Table 1:** Clinical information of HNSCC patients in The Cancer Genome Atlas.

	Primary tumor, N = 500	Tissue normal, N = 44	p
Age:			0.251
≤ 60	244 (48.9%)	17 (38.6%)	
> 60	255 (51.1%)	27 (61.4%)	
Alcohol exposure:			0.096
No	157 (31.4%)	19 (43.2%)	
Yes	332 (66.4%)	23 (52.3%)	
Not Reported	11 (2.20%)	2 (4.55%)	
Tumor stage:			
stage I/II	95 (19.00%)	–	
stage III/I	337 (67.40%)	–	
Not Reported	68 (13.6%)	–	
Pathologic T:			.
T0	1 (0.20%)	–	
T1	45 (9.00%)	–	
T2	132 (26.4%)	–	
T3	96 (19.2%)	–	
T4	11 (2.20%)	–	
T4a	156 (31.2%)	–	
T4b	4 (0.80%)	–	
TX	33 (6.60%)	–	
Not Reported	22 (4.40%)	–	
Pathologic N:			
N0	171 (34.2%)	–	
N1	65 (13.0%)	–	
N2	12 (2.40%)	–	
N2a	7 (1.40%)	–	
N2b	101 (20.2%)	–	
N2c	44 (8.80%)	–	
N3	7 (1.40%)	–	
NX	69 (13.8%)	–	
Not Reported	24 (4.80%)	–	
Pathologic M:			
M0	186 (37.2%)	–	
M1	1 (0.20%)	–	
MX	61 (12.2%)	–	
Not Reported	252 (50.4%)	–	
Lymphovascular invasion:			
No	219 (43.8%)	–	
Yes	120 (24.0%)	–	
Not Reported	161 (32.2%)	–	
subtype:			
Atypical	68 (13.6%)	–	
Basal	85 (17.0%)	–	
Classical	49 (9.80%)	–	
Mesenchymal	75 (15.0%)	–	
Not Reported	223 (44.6%)	–	

### M6A RNA Methylation Regulator Identification

The data on 21 m6A regulators were collected from the document (14). TCGA-HNSCC dataset included data on 500 HNSCC and 44 normal tissues. After performing logarithmic transformation and normalization of the expression data of all samples, the differences in expression of m6A regulators in tumor tissues versus normal tissues were illustrated *via* heatmaps.

### Copy Number Variant and Single Nucleotide Variant Mutation Analysis

The R package “maftool” was used for SNV analysis, with default settings used for all parameters (19). For the TCGA dataset, SNV and CNV amplifications and deletions were determined and summarized for each gene in all samples and each cancer type; the R package “ComplexHeatmap” was then used to generate SNV mutation heatmaps for each cancer type (20). In the visual display of the Cancer Cell Line Encyclopedia (CCLE) and Genomics of Drug Sensitivity in Cancer (GDSC) datasets, CNV data included gene-level circular binary segmentation (CBS) copy number data and was not processed using the Gistic2 number algorithm.

### Kyoto Encyclopedia of Genes and Genomes \ and Gene Ontology \ Enrichment Analyses

The R package “clusterProfiler” was used to conduct KEGG pathway and GO enrichment analyses (21). The parameters used were pAdjustMethod = “BH” and p-value cutoff = 0.05. Then, Cytoscape cluGo was used to visualize KEGG channels and GO networks.

### Construction of the Risk Signature

The selection of candidate risk m6A regulators related to genes was performed *via* univariate Cox regression and Lasso analyses. Multivariate Cox regression analysis was used to establish a profile of independent prognostic genes. The risk score calculation was conducted according to the following formula: $R_{i}=\Sigma_{j=1}^{n}\beta_{j}x_{ij},$ where *R_i_* represents the risk rate of the first sample; *I* = 1, 2,…, 500; *β_j_* denotes the regression coefficient of the gene *j* in the Cox model; *j* = 1, 2,…, 12; and *x_ij_* indicates the expression value of the gene *j* in sample *i*. The patients were divided into high-risk and low-risk groups, based on the mean value of the risk score.

### Prediction of M6A Methylation Modification Sites

The m6A modification sites were predicted using the sequence-based RNA adenosine methylation site predictor (SRAMP) software. As a public prediction server, SRAMP combines three random forest classifiers that exploit the positional nucleotide sequence pattern, the K-nearest neighbor information, and the position independent nucleotide pair spectrum features to accurate identification of RNA m6A sites. SRAMP uses either genomic sequences or cDNA sequences as its input, achieving competitive performance in cross-validation tests and rigorous independent benchmarking tests (22). The gene symbols of significantly enriched genes were converted into gene bank Accession Number IDs by utilizing the “bitr” function of the R package “Clusterprofiler.” Then, the R package “rentrez” was used to download the transcript sequence of each gene from the gene bank (2021.3.6 download) in the same gene selection database. The longest transcript in the database of the same gene was selected.

### Immune Infiltration

Tumor immune infiltration analysis was based on the proportion of immune cells predicted by using the Cibersort software. First, the death risk of each TCGA-HNSCC sample was calculated according to the 12-gene multivariate Cox model screened *via* Lasso analysis. Then, 500 HNSCC cancer tissue samples were divided into high- and low-risk groups, based on the median value of the death risk of all samples. Cibersort was used to estimate the infiltration ratios of 22 immune cells in 500 HNSCC cancer tissue samples. A visualization method (bar chart) was used to show differences in immune cell infiltration in the high- and low-risk groups, and the Wilcoxon test was further used to perform statistical tests for the differences in immune cell infiltration between these groups. For immune cells with significant differences between the high- and low-risk groups, all samples were divided into high- or low-infiltration groups using median or tertile values; the survival difference between the two groups of patients was then analyzed using the R package “survival” (23).

### Relationships between Immune Score, Stromal Score, and Tumor Mutational Burden (TMB)

The ESTIMATE algorithm (Estimation of STromal and Immunecells in MAlignant Tumor tissues using Expression data) (24) was used to value stromal and immune microenvironment infiltration based on gene transcriptome data. The analysis method is integrated with the R package “ESTIMATE.” TMB is a measure of the total number of mutations per megabyte of tumor tissue. It represents the mutation density of tumor genes and defined as the average number of mutations in the tumor genome including the total number of gene coding errors, base substitution insertions, or deletions (25).

### Immune Response Analysis

The IMvigor210CoreBiologies and GSE78220 datasets were used to analyze the correlation between prognostic signature and the treatment response to ICIs, including differences in effectiveness and survival prognosis. The ICI efficacy was evaluated according to the response evaluation criteria in solid tumors (RECIST) v.1.1 standards. Responders are referred to as patients with objective response rate (ORR), complete remission (CR), or partial remission (PR), whereas non-responders are defined as those with stable disease (SD) or progressive disease (PD).

### Statistical Analysis

**Figure 1:**
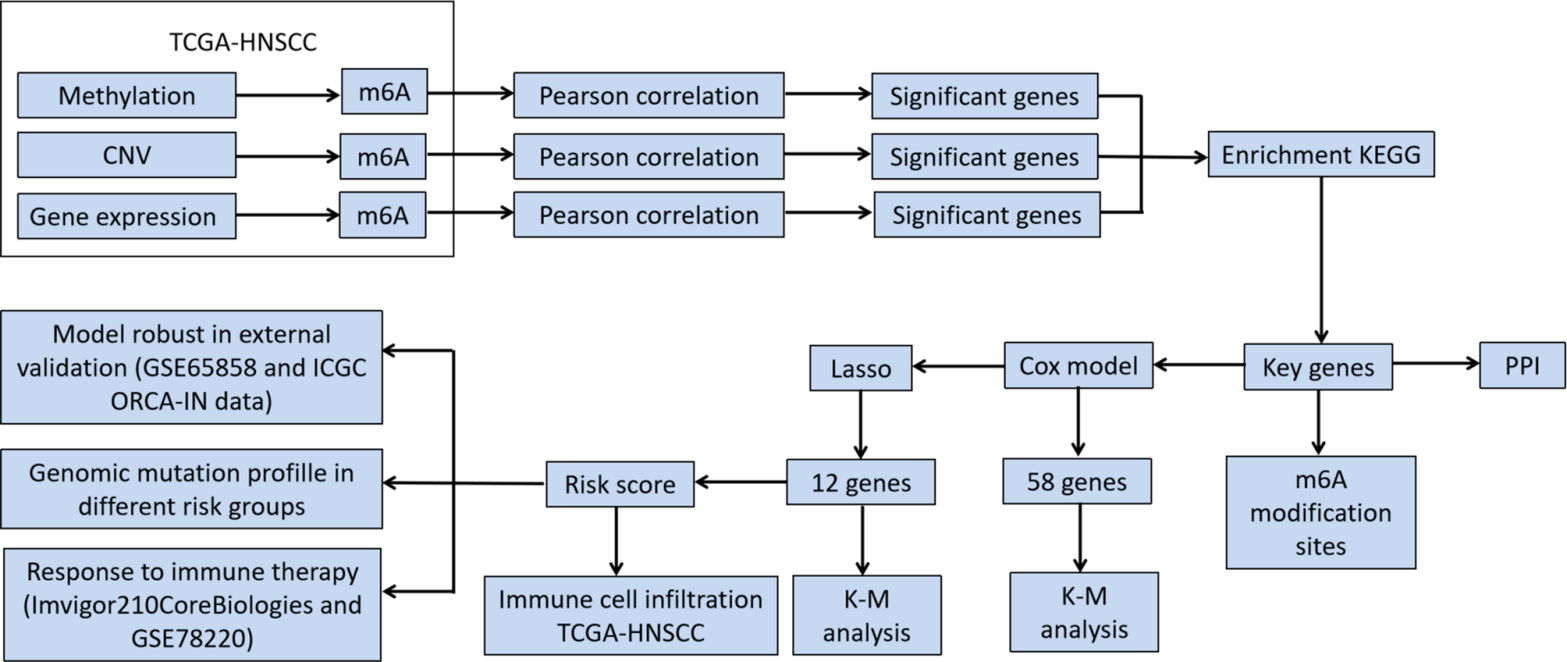
Flowchart of the study.

A flow chart for the m6A regulator-related genes signature development and subsequent analyses has been provided in **Figure 1**. Pearson’s correlation coefficients and the corresponding *p* values were calculated between m6A regulatory factors and other genes at three levels, namely transcription level, methylation level, and CNV mutation number. Pearson’s correlation coefficients of >0.5 or <-0.5 and a *p*-value of <0.05 were used as thresholds to perform screening of correlations at the three levels of gene expression, methylation, and CNV, respectively. Genes with significant correlations at all three levels were selected for the next step of the analysis.

Univariate and multivariate Cox regression analyses were performed to analyze the correlations between the genes and the overall survival (OS) in HNSCC patients. First, significant m6A regulator-related genes were screened according to the univariate Cox regression results; they were then further screened using Lasso regression analysis. The significantly different genes screened in the above-mentioned step were used to establish a multivariate Cox model. This was combined with Lasso regularization to conduct variable screening. The Lasso screening parameters were α = 1, nλ = 100, and λ=λ.min. Those genes whose multivariate Cox regression coefficients were not equal to zero under Lasso regularization were selected as target genes to conduct the next step of the analysis. Kaplan–Meier (K-M) and log-rank analyses were used to evaluate the survival differences between patient groups. Results were considered statistically significant if a two-sided *p-*value <0.05.

## Results

### Alteration of M6A RNA Methylation Regulators in TCGA-HNSCC Patients

**Figure 2:**
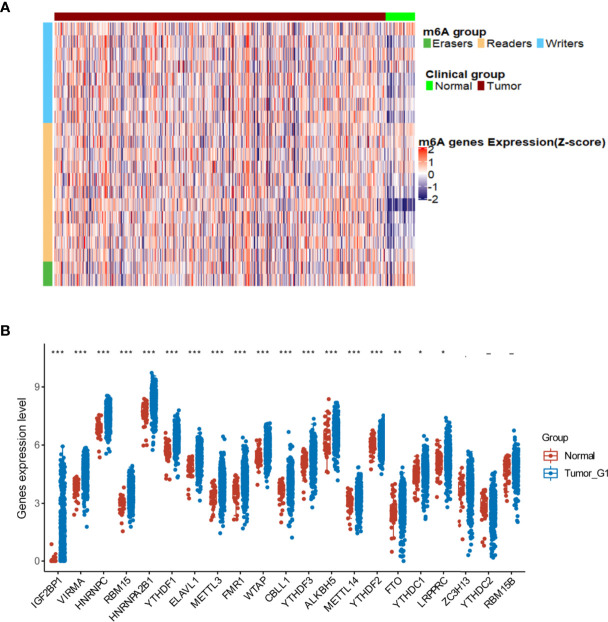
Alterations in m6A regulators in The Cancer Genome Atlas- head and neck squamous cell carcinoma (HNSCC) patients. **(A)** Heatmap showing the expression of m6A regulators in tumor and normal tissues. **(B)** Differences illustrated in expression levels between HNSCC (blue box) and normal (red box) tissues for 21 m6A regulators. For t-test: ns, *p* ≥ 0.05; *p < 0.05; **p < 0.01; ***p < 0.001.

In total, 544 cases based on TCGA-HNSCC transcriptomic data were analyzed, including 500 tumor tissues and 44 normal tissue samples, revealing that the expression of 18 m6A regulator genes was higher in tumor tissues than that in normal tissues, including 7 m6A writer genes (*VIRMA, RBM15, METTL3, WTAP, CBLL1, METTL14*, and *LRPPRC*), 9 reader genes (*IGF2BP1, HNRNPC, HNRNPA2B1, YTHDF1, ELAVL1, FMR1, YTHDF3, YTHDF2*, and *YTHDC1*) and 2 erasers genes (*ALKBH5* and *FTO*) (**Figures 2A, B**). These results indicate that m6A modification is related to the occurrence of HNSCC.

### Identification of M6A Regulator-Related Genes in HNSCC Patients

**Figure 3:**
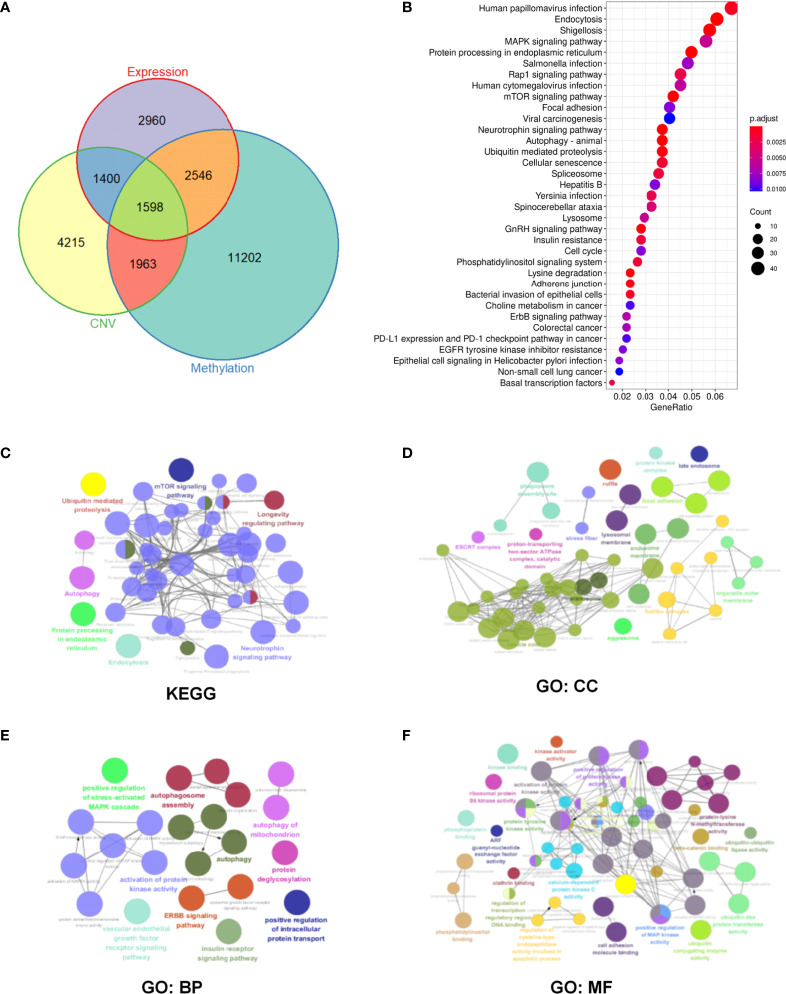
Identification and enrichment pathway analysis of m6A regulator-related mRNAs in head and neck squamous cell carcinoma (HNSCC) patients. **(A)** Overlapped differentially expressed genes at CNV, methylation, and transcription levels. **(B)** Enrichment analysis of 1598 m6A regulator-related genes *via* the Kyoto Encyclopedia of Genes and Genomes (KEGG) pathway. **(C–F)** Significantly enriched KEGG and Gene Ontology (GO) pathways. Objects with the same color belong to the same group and have been depicted using the same color label on the side. GO analysis helped classify regulators into the biological process (BP), cellular component (CC), and molecular function (MF) groups.

Pearson correlation analysis was used to perform screening of m6A regulator-related genes with significant correlations at the CNV, mutation, and transcription levels (|Pearson’s R| > 0.5 and *p* < 0.05). As a result, 9176 genes were detected and found to be significantly correlated with m6A regulatory genes at the CNV level (**Table 2**); 17,309 genes were significantly correlated at the methylation level (**Table 3**), and 8504 genes were significantly correlated at the transcription level (**Table 4**). The Venn diagram presented in **Figure 3A** shows that the expression of 1598 genes at the transcription, CNV, and methylation levels were related to the expression of m6A regulators. The specific 1598 genes are shown in **Supplementary Table 2**. Among them, 192 genes were significantly correlated with *YTHDC2* at the methylation level, 146 genes were correlated with *YTHDC2* at the CNV level, and 796 genes were significantly correlated with *YTHDC2* at the transcription level. Thirty-three genes were significantly correlated with *YTHDC2* at the methylation, CNV, and transcription levels. The parts of genes that are highly correlated with m6A regulatory factors are summarized in **Supplementary Figure 1**.

**Table 2:** Genes were significantly correlated with m6A regulators at the level of copy number variation (top 20).

Correction	adjust_P	gene_m6A	Gene	Type
1	0	YTHDF2	TAF12	CNV
1	0	YTHDF2	RAB42	CNV
1	0	YTHDF2	RNU11	CNV
1	0	YTHDF2	GMEB1	CNV
1	0	YTHDF2	SCARNA24	CNV
1	0	RBM15	AHCYL1	CNV
1	0	RBM15	STRIP1	CNV
1	0	RBM15	ALX3	CNV
1	0	RBM15	UBL4B	CNV
1	0	RBM15	SLC6A17	CNV
1	0	RBM15	KCNC4	CNV
1	0	RBM15	SNORA25	CNV
1	0	LRPPRC	PLEKHH2	CNV
1	0	LRPPRC	RN7SKP66	CNV
1	0	LRPPRC	DYNC2LI1	CNV
1	0	LRPPRC	ABCG5	CNV
1	0	LRPPRC	ABCG8	CNV
1	0	RBM15B	MANF	CNV
1	0	RBM15B	RBM15B	CNV

**Table 3:** Genes were significantly correlated at the methylation level (top 20).

Correction	adjust_P	gene_m6A	Gene	Type
0.85	4.67E-148	ZC3H13	COL10A1	Methylation
0.84	2.39E-142	FTO	BCAS3	Methylation
0.83	2.09E-134	ZC3H13	PNISR	Methylation
0.82	3.22E-132	METTL14	LARP7	Methylation
0.82	4.50E-132	FTO	WDR25	Methylation
0.82	1.79E-130	ZC3H13	CEP57L1	Methylation
0.82	8.15E-130	ZC3H13	ZNF326	Methylation
0.82	2.39E-129	ZC3H13	OSMR	Methylation
0.82	2.43E-129	ZC3H13	PRPF39	Methylation
0.82	2.94E-129	FTO	PRKCE	Methylation
0.82	3.16E-129	ZC3H13	SNX14	Methylation
0.82	4.15E-129	ZC3H13	WRN	Methylation
0.82	3.48E-127	METTL14	COL10A1	Methylation
0.82	3.88E-127	ZC3H13	PPIP5K2	Methylation
0.82	8.70E-127	METTL14	USP38	Methylation
0.81	2.08E-126	ZC3H13	NUF2	Methylation
0.81	3.05E-126	ZC3H13	ABHD13	Methylation
0.81	6.68E-126	ZC3H13	EXOC5	Methylation
0.81	1.58E-125	ZC3H13	RUFY2	Methylation
0.81	2.84E-125	METTL14	RCHY1	Methylation

**Table 4:** Genes were significantly correlated with m6A regulators at the transcription level (top 20).

Correction	adjust_P	gene_m6A	Gene	Type
0.93	1.74E-211	YTHDF3	VCPIP1	Expression
0.91	9.12E-191	ZC3H13	AKAP11	Expression
0.91	6.60E-189	ZC3H13	LRCH1	Expression
0.90	1.25E-180	RBM15B	QRICH1	Expression
0.90	1.24E-178	KIAA1429	MTDH	Expression
0.89	9.93E-175	LRPPRC	CEBPZ	Expression
0.89	2.66E-173	KIAA1429	KIAA0196	Expression
0.89	4.53E-170	YTHDF3	ARFGEF1	Expression
0.89	2.54E-169	ZC3H13	VWA8	Expression
0.88	4.07E-167	KIAA1429	YTHDF3	Expression
0.88	4.07E-167	YTHDF3	KIAA1429	Expression
0.88	4.98E-167	ZC3H13	UTP14C	Expression
0.88	2.10E-166	ZC3H13	PROSER1	Expression
0.88	5.66E-160	RBM15B	WDR82	Expression
0.88	1.01E-159	LRPPRC	WDR43	Expression
0.88	1.79E-159	KIAA1429	UBR5	Expression
0.88	2.55E-159	ZC3H13	ELF1	Expression
0.87	1.02E-155	KIAA1429	VCPIP1	Expression
0.87	1.24E-155	YTHDC1	CENPC	Expression

### KEGG and GO Enrichment Pathway Analysis of M6A Regulator-related genes

Based on 1598 m6A regulator-related genes, KEGG pathway enrichment analysis was conducted and revealed that 65 pathways were significantly enriched (*p <*0.05) (**Supplementary Table 3**). The top 35 enrichment KEGG pathways have been shown in **Figure 3B**. Furthermore, 187 m6A regulator-related genes in the top ten pathways were selected for subsequent analysis (**Supplementary Tables 4** and **5**). The Cytoscape plug-in “ClueGO” was used to perform network analysis using the KEGG pathway and GO results of these 187 m6A regulator-related genes. In terms of KEGG pathways, mTOR, autophagy, and the ubiquitin-mediated proteolysis signaling pathway were found to be significantly enriched (**Figure 3C**). In the cellular component (CC), focal adhesion and the protein kinase complex were found to be significantly enriched (**Figure 3D**). For the biological process (BP), significantly enriched pathways included protein kinase-related processes, autophagy, and the stress-activated mitogen-activated protein kinase (MAPK) cascade (**Figure 3E**). For molecular function (MF), protein kinase activity and cell adhesion were found to be significantly enriched (**Figure 3F**).

Moreover, analysis of the SNV and CNV mutations of these 187 m6A regulator-related genes in head and neck cancer, ovarian cancer, cervical cancer, endometrial cancer, colorectal cancer, and breast cancer showed that endometrial cancer presented with the most considerable mutation burden for SNV, whereas the mutation burden in HNSCC was relatively low (**Supplementary Figure 2**).

### Screening Key Genes Related to M6A in HNSCC

**Figure 4:**
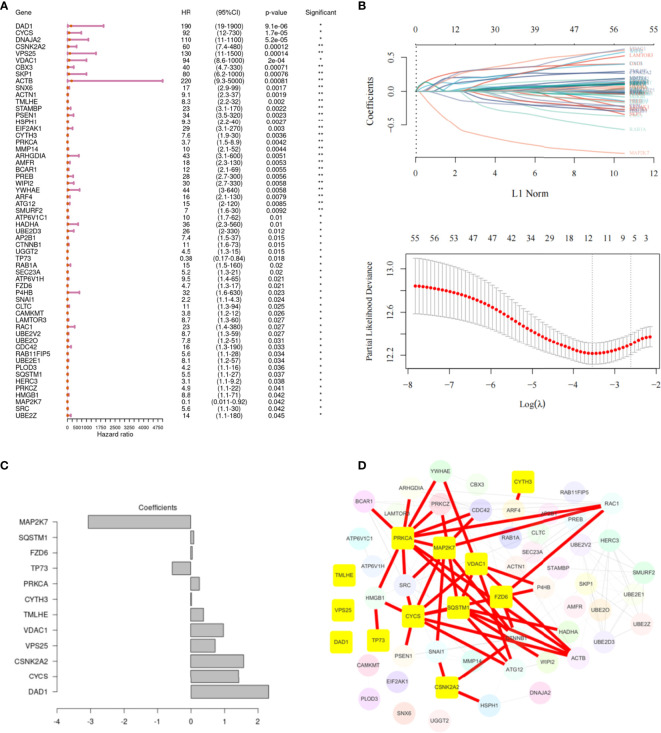
Screening of the key genes related to m6A in head and neck squamous cell carcinoma (HNSCC). **(A)** Univariate Cox regression analysis, showing 58 m6A regulator-related genes associated with the survival of patients with HNSCC. **(B)** The 12 m6A regulator-related genes are most relevant to the survival status, as identified by screening with the Lasso–Cox regression. The partial likelihood deviance is shown against log (Lambda). A vertical line is drawn at the value fitting the tenfold cross-validation. **(C)** The Lasso–Cox regression coefficients for the 12 identified genes. **(D)** PPI network of 58 genes, highlighting the top 12 genes.

**Figure 5:**
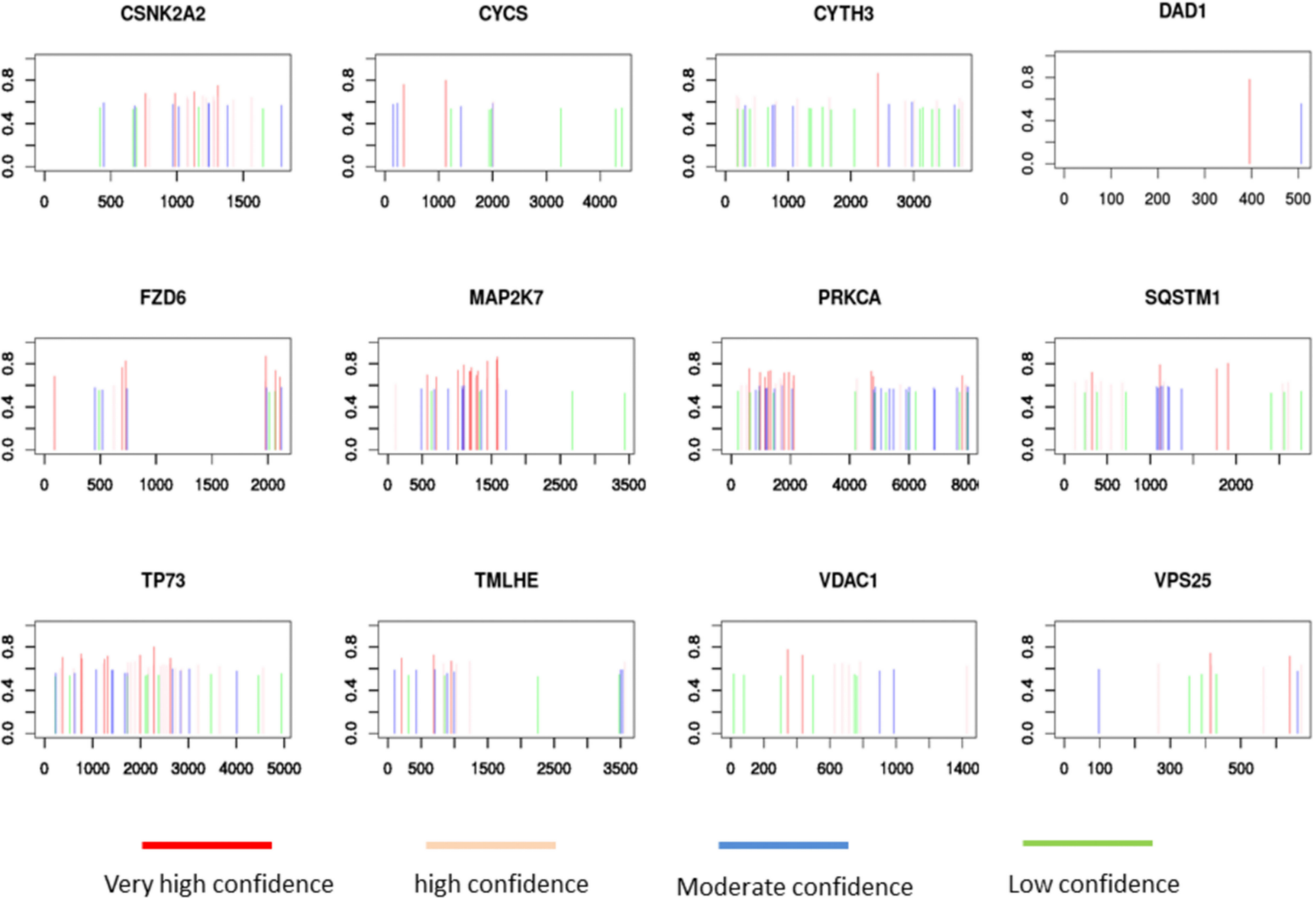
SRAMP analysis of potential m6A methylation modification sites on 12 m6A regulator-related genes.

Univariate Cox regression analysis showed that 58 m6A regulator-related genes were associated with the survival of HNSCC patients (**Figure 4A**). The K-M survival curve of patients with high- and low-expression groups of certain key m6A regulator-related genes is presented in **Supplementary Figure 3**. Subsequently, HNSCC patients were divided into low- and high-expression groups according to the median expression of each m6A regulator-related gene. Lasso-Cox regression results indicated that 12 genes could be considered as prognostic factors of OS in HNSCC patients (**Figure 4B**). The lasso-Cox regression coefficients for these 12 genes have been shown in **Figure 4C**. Construction of a protein–protein interaction (PPI) network of 58 genes using the string protein interaction database and revelation of the top 12 genes indicated that *PRKCA, MAP2K7, VDAC1, FZD6, SQSTM1*, and *CYCS* were located at key positions (**Figure 4D**). Moreover, the 12 key m6A regulator-related genes revealed by using the SRAMP software were considered to be potential m6A methylation modification sites (**Figure 5**).

### Construction and Evaluation of an M6A Regulator-Related Genes’ Signature

**Figure 6:**
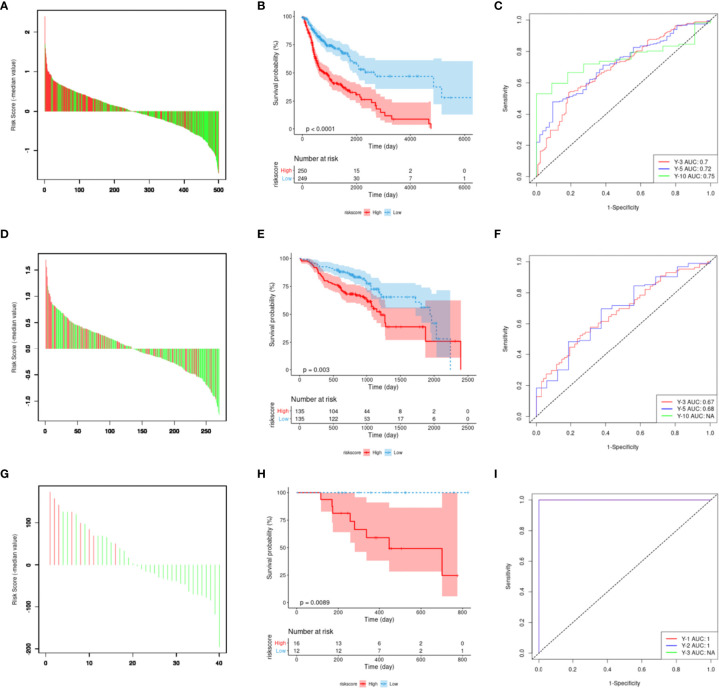
Construction and evaluation of m6A regulator-related mRNA signature. **(A)** The risk score of the 12-gene model in the Cancer Genome Atlas dataset. Red represents the time to death, green represents survival status, and the ordinate represents the difference between risk and median scores. **(B)** Model risk score prognostic analysis of patient survival. **(C)** The receiver operating characteristic (ROC) curve is predicted by using the model. **(D–F)** External verification using the GSE65858 dataset. **(G–I)** Verification result for the ICGC ORCN-IN dataset.

The 12 genes selected *via* Lasso regression analysis were used for multivariate Cox regression analysis to obtain the survival risk score of each sample in TCGA-HNSCC (**Figure 6A**). Then, risk scores were calculated for every cancer sample calculated by using the following formula: Risk_raw_ = DAD1*2.316+CYCS*1.426+CSNK2A2*1.576+VPS25*0.728+VDAC1*0.976+TMLHE*0.381+CYTH3*0.024+PRKCA*0.260-TP73*0.567+FZD6*0.047+SQSTM1*0.094-MAP2K7*3.070. It is normalized while visualizing the risk score using the following formula: Risk score = Risk_raw_-median of all Risk_raw_. Based on the median signature risk score, the patients were divided into high- and low-risk groups. The K-M survival curve showed that high-risk patients exhibited poorer OS than low-risk patients based on TCGA-HNSCC data (*p <*0.001; **Figure 6B**). Patients’ 3-, 5-, and 10-year survival statuses were assessed using the risk rate; the prediction accuracy was evaluated using the area under the curve (AUC). The predicted AUC values for survival at 3, 5, and 10 years were 0.70, 0.72, and 0.75, respectively (**Figure 6C**). Multivariate analyses confirmed that the high-risk group was an independent inferior factor for OS (HR=3.00, 95% CI: 2.00**–**4.60, p < 0.001), after adjusting gender, age, clinical stage, and histology grade (**Table 5**).

**Table 5:** The multivariate analyses of overall survival according to risk group, after adjusting for other potential predictors in TCGA.

Characteristics	HR	95%CI	p-value
Age (years)			
>60 vs. ≤60	1.10	0.81-1.60	0.44
Gender			
Male vs. Female	0.81	0.56-1.20	0.25
Clinical stage			
III/IV vs. I/II	1.00	(0.73-1.40)	0.87
Histology grade			
G3/G4 vs. G1/G2	1.20	0.84-1.80	0.29
Risk group			
High vs. Low	3.00	(2.00-4.60)	<0.001

The prognostic values of the signature scores were externally verified using the GSE65858 dataset, which revealed that there was a significant difference in OS between the high- and low-risk groups (*p <*0.001; **Figures 6D, E**). The predicted AUC values for 3- and 5-year survival based on the signature score were 0.67 and 0.68, respectively (**Figure 6F**). These values were slightly lower than the predicted performance values based on the training set. This risk factor could not predict the 10-year survival status for the GSE65858 dataset, mainly because the follow-up times of the cases in the GSE65858 data were lower than 10 years (**Figure 6F**).

During the external verification of the ICGC ORCA-IN dataset, the OS of high-risk patients was significantly worse than low-risk patients (*p* = 0.0089; **Figures 6G, H**). Additionally, the predicted AUC values of the 1- and 2-year survival statuses were both estimated to be 1. The follow-up time of cases in this dataset was not sufficient to predict survival rates >3 years (**Figure 6I**).

### Comparison of Mutation Status Between High- and Low-Risk Groups

**Figure 7:**
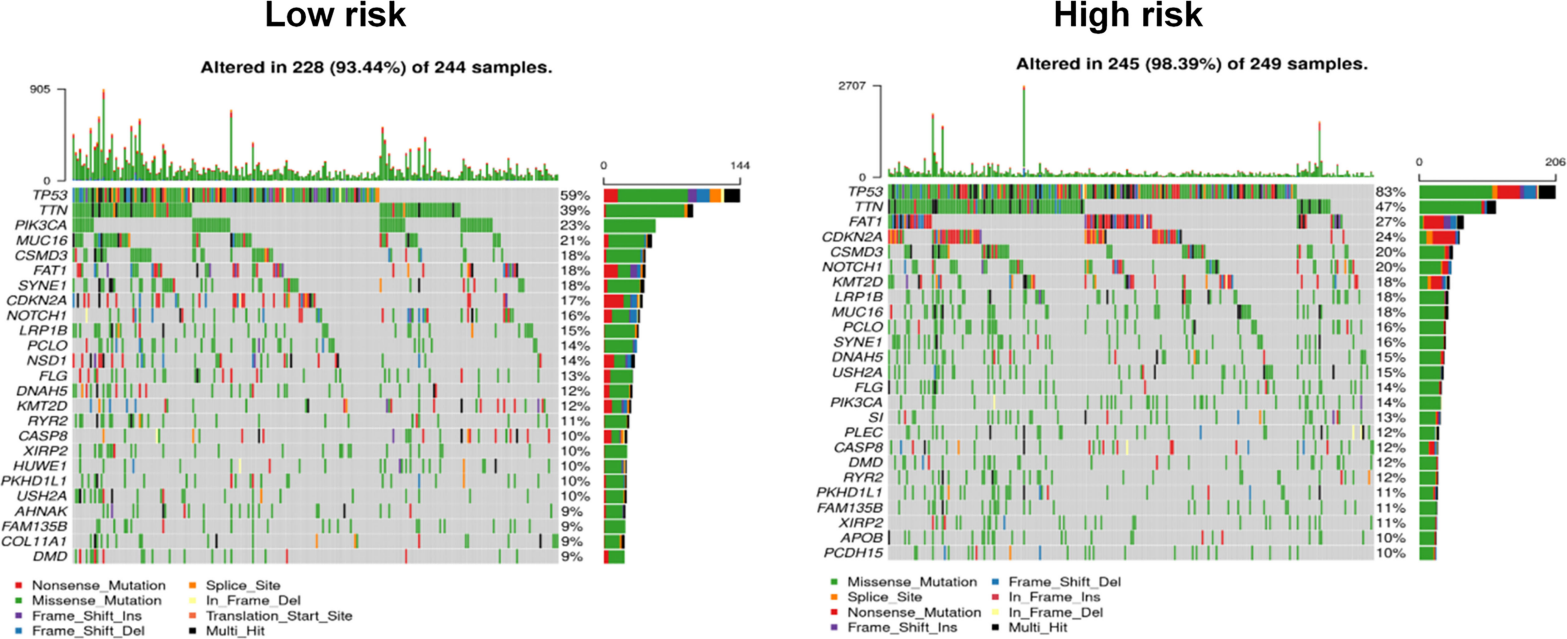
Comparison of mutational differences between high- and low-risk groups.

Gene mutations are considered the main driving factors for the occurrence and development of cancer. To better understand the underlying mechanism by which the signature of the risk score could be used to effectively assess the prognoses of patients, the gene mutations of patients in the high- and low-risk groups were investigated. As shown in **Figure 7**, using the model, it was predicted that the overall mutation rate of the low-risk group was ~5% lower than that of the high-risk group (89.44% vs. 98.39%, respectively). Additionally, the two groups of high-frequency mutation genes and mutation rates also showed significant differences. In the low-risk group, the five genes with the highest mutation rates were TP53 (59%), TTN (39%), PIK3CA (23%), MUC16 (18%), and CSMD3 (18%), while the five genes with the highest mutation rates in the high-risk group were TP53 (83%), TTN (47%), FAT1 (27%), CDKN2A (24%), and CSMD3 (20%). In addition, weak associations between the risk score and TMB (r = 0.11, *p* = 0.007) are shown in **Supplementary Figure 4A**.

### Association Between High-Risk Score and Low Infiltration of Anti-Tumor Immune Cells

**Figure 8:**
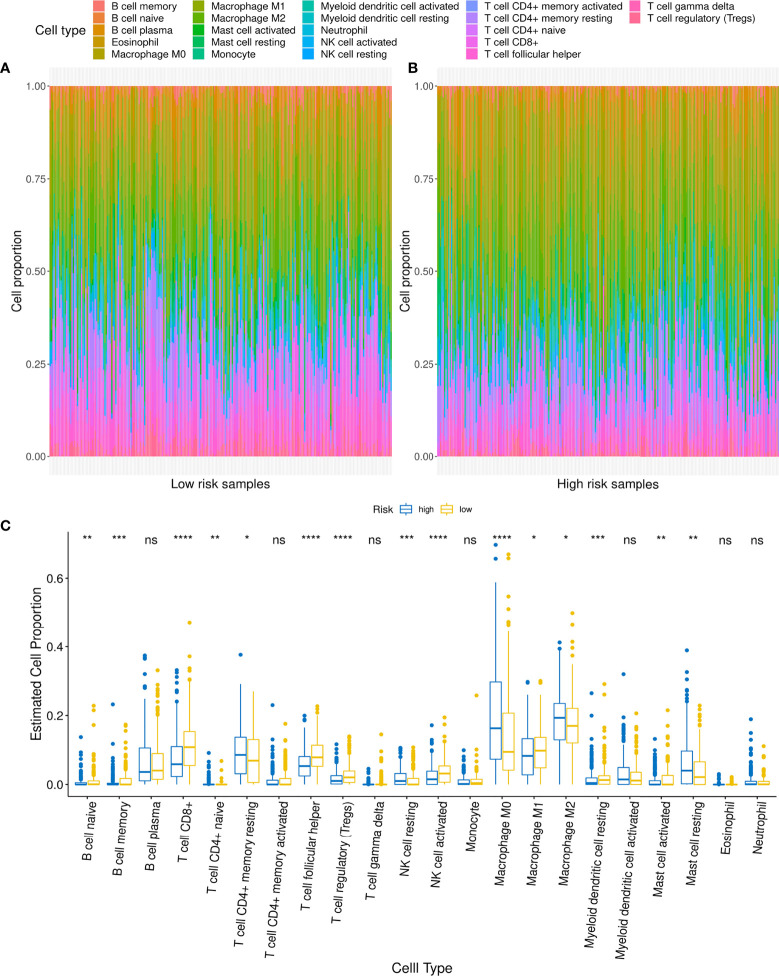
Relationship between low- and high-risk groups and immune cell infiltration. **(A, B)** Comparison of distribution of immune cell infiltration types in low- and high-risk groups. **(C)** Difference in infiltration of various immune cells between low- and high-risk groups. For t-test: ns, *p* ≥0.05; *p < 0.05; **p < 0.01; ***p < 0.001; ****p < 0.0001.

Considering that patients in the different risk groups demonstrated different prognoses and molecular variation patterns, it could be suggested that the risk score might also be related to the infiltration of immune cells in the tumor microenvironment. First, we found that the risk score was weakly associated with the immune score (r = -0.15, *p* = 0.0008) and stromal score (r = 0.12, *p* = 0.01) (**Supplementary Figures 4B, C**). Then, immune cell infiltration was compared between the high- and low-risk groups using Cibersort, with findings revealing that eight types of immune cell infiltration showed highly significant differences between the two groups (*p <*0.001) among the 22 types of immune cell infiltration, including macrophage M0, B cell memory, natural killer (NK) cell activated, NK cell resting, T cell CD8, T cell regulatory (Tregs), T cell follicular helper, and myeloid dendritic cell resting (**Figures 8A, B**). Among these, the proportions of T cell CD8+, T cell regulatory, and T cell follicular helper cells were significantly higher in the low-risk group than those observed in the high-risk group. Macrophage M0 cell infiltration was significantly higher in the high-risk group than that in the low-risk group (**Figure 8C**).

Considering the characteristics of m6A-related genes and immune cell TCGA-related data, we analyzed the correlation between the expression of m6A regulators and the expression of PD-L1. The results showed that 19 m6A regulator expression were related to PD-L1 expression. Among them, three genes with the strongest correlation coefficients with PD-L1 expression were YTHDC2 (R=0.388), YTHDF3 (R=0. 0.273) and METTL14 (R=0.271) (**Supplementary Figures 5A–C**), and the correlation between other m6A methylation regulators and PD-L1 is shown in the **Supplementary Table 6**. Furthermore, analyzed the expression of PD-L1 in the high- and low-risk groups of patients, and the results showed that there was no significant difference in the expression of PD-L1 between the two groups (**Supplementary Figure 5D**).

### Ability of High-Risk Score to be Used for Prediction of Poor Responses to ICIs

**Figure 9:**
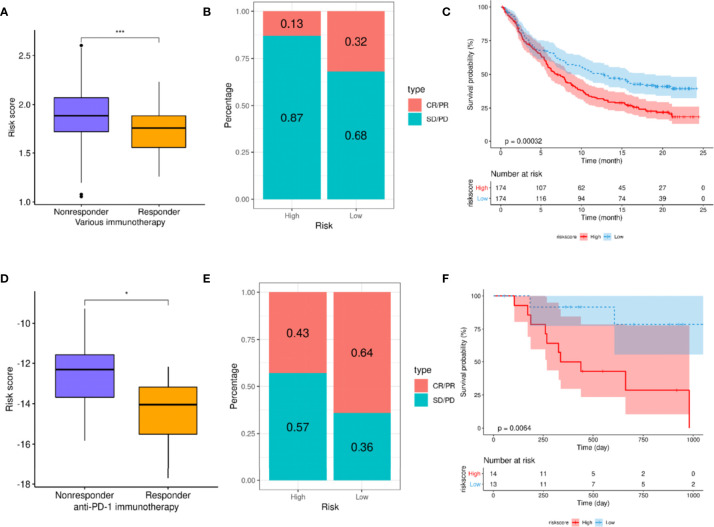
A high-risk score predicts poor response to immune checkpoint inhibitors (ICIs). **(A–C)** Exploration of the utility of risk scores as prognostic markers for ICIs using the Imvigor210CoreBiologies dataset. **(A)** Comparison of risk scores between different immune response states. **(B)** Comparison of immune response ratio between high- and low-risk groups. **(C)** Overall survival (OS) analysis of high- and low-risk groups. **(D, E)** Verification of relationship between risk score and immune response using the GSE78220 dataset. **(D)** Comparison of risk scores between different immune response groups. **(E)** Comparison of immune response rates between high- and low-risk groups. **(F)** Analysis of the OS of patients in high- and low-risk groups. For t-test: *p < 0.05; ***p < 0.001.

Considering that patients in the high- and low-risk groups exhibited different levels of immune cell infiltration, it could be suggested that these groups might demonstrate an impression of the efficacy of ICIs. As there is currently no transcriptome data available for patients receiving ICIs in HNSCC, data reported for other cancers were used to verify this speculation. The results based on the use of the Imvigor210CoreBiologies dataset showed that non-responders to ICIs (SD + PD) presented with higher risk scores than responders (CR + PR; *p <*0.001; **Figure 9A**). Concurrently, the ORR was significantly higher in the low-risk group than that in the high-risk group (32% vs. 13%, respectively; **Figure 9B**). Additionally, patients in the high-risk group exhibited significantly adverse OS compared to those in the low-risk group (*p* = 0.00032; **Figure 9C**). This finding indicates that the risk score can be used as a prognostic marker of the immune response.

Finally, the GSE78220 dataset was used to verify the relationship between risk score and immune response, revealing that the risk scores of the non-responders were significantly higher than those of the responders (*p <*0.001; **Figure 9D**). Moreover, the ORR in the low-risk group was significantly higher than that in the high-risk group (64% vs. 43%, respectively; **Figure 9E**). Simultaneously, the OS of the low-risk group was significantly better than that of the high-risk group (*p* = 0.0064; **Figure 9F**).

## Discussion

Owing to the heterogeneity of HNSCC and the unsatisfactory therapeutic effects encountered, it is necessary to determine an accurate prognosis for HNSCC patients to explore and establish proper individualized treatments (26). Previous studies have indicated that the abnormal expression of m6A regulators may be related to the occurrence and development of various cancers, including breast cancer, bladder cancer, glioma, and colorectal cancer (9, 15). However, recent research into the pathological role of m6A regulatory factors in HNSCC progression remains limited, and few studies have addressed prognostic and predictive evaluation biomarkers for immunotherapy using m6A-related genes. The findings of this study showed that the expression of certain m6A regulators was upregulated in HNSCC tissues compared to normal tissues. This study also identified 1598 m6A regulator-related genes that were significantly related to CNV, methylation, and transcription levels. KEGG pathway enrichment analysis based on m6A regulator-related genes helped identify 187 genes in the top ten pathways; 12 genes were identified as prognostic factors *via* Lasso regression analyses. Subsequently, a prognostic signature constructed using these 12 m6A regulator-related genes was used to better distinguish between outcomes in HNSCC. Interestingly, this signature was associated with immune cell infiltration and with the responses and outcomes pertaining to therapy with ICIs.

Here, the expression of various m6A RNA methylation regulators was different in tumor and normal tissues within the TCGA-HNSCC database; this was consistent with the findings of previous reports (27, 28). M6A is known to demonstrate complex functions and is widely involved in regulating mRNA splicing, translation, decay, 3’-end processing, and non-coding RNA processing (29, 30). Through these processes, it demonstrates the biological function of methylation modification. Previous studies have demonstrated that m6A regulators related to mRNA signatures can exhibit an independent prognostic value, such as those of pancreatic and bladder cancer (31, 32). Here, 1598 m6A regulator-related genes were found to be significantly correlated to CNV, methylation, and transcription levels. A prognostic signature for HNSCC patients was then developed based on 12 m6A regulator-related genes *via* Lasso regression analyses. SRAMP software analysis showed that the 12 genes in this prognostic signature possessed m6A methylation modification sites, indicating that m6A methylation might affect the expression of these 12 genes. Furthermore, the findings of this study showed that the developed signature could be used to effectively distinguish between the prognoses for TCGA-HNSCC patients; the prognostic evaluation value of the signature was also verified in two other datasets (GSE65858 and ICGC ORCA-IN). Notably, the AUC of the developed prognostic signature demonstrated high accuracy for the training set and the two validation sets (GSE65858 and ICGC ORCA-IN). These results indicate that the signature demonstrates marked clinical availability.

Previous reports have shown that key genes within this prognostic signature are related to the occurrence and development of tumors. For example, the following six genes are located at the center of the key protein interaction network: PRKCA, MAP2K7, VDAC1, FZD6, SQSTM1, and CYCS. PRKCA is overexpressed in oral tongue squamous cell carcinoma and has been associated with poor prognosis (33). MAP2K7A has been used as a candidate tumor suppressor in gastric cancer (34) and T-cell acute lymphoblastic leukemia (35, 36), and has demonstrated the potential to be used as a prognostic biomarker for prostate cancer (37). The deletion of VDAC1 has been shown to cause glioblastoma (GBM) metabolism rewiring, which can, in turn, affect epigenetic modification and inhibit tumor development and progression (38). It has also been revealed that the FZD6-fibronectin actin axis can be exploited in drug development for highly metastatic forms of breast cancer (39). The pleiotropic protein p62/SQSTM1 is subjected to degradation during autophagy, and its expression has been demonstrated to increase in primary HNSCC tumors. CYCS encodes the core component protein of the mitochondrial electron transport chain, which is also involved in the initiation of apoptosis and various tumor processes (40, 41). Although the mechanisms of some of these genes in HNSCC were not clear in the present study, studies have also confirmed that they could be involved in the occurrence and development of tumors in other cancers. The biological functions of these key genes in HNSCC are worthy of comprehensive *in vivo* and *in vitro* studies.

The tumor microenvironment plays a vital role in the occurrence and development of tumors, especially in the immune infiltration of tumor cells (42, 43). This not only affects the development of tumors but also affects the efficacy of ICIs (44). Here, a significant difference was found in the level of immune cell infiltration between the high- and low-risk groups; patients in the high-risk group exhibited greater infiltration of macrophages and less extensive infiltration of CD8+ T cells. The degree of CD8+ T cell infiltration has been established to be positively associated with better prognoses and immunotherapy effects for patients (45, 46). Considering that immune cell infiltration is an important factor in predicting the treatment effect of ICIs, the original aim of this study was to analyze the predictive value of risk score in HNSCC patients using ICI treatments. Although RNA sequencing or gene expression microarray data for ICI treatments were lacking regarding HNSCC, public transcription data from urothelial carcinoma and melanoma patients were used to confirm that high-risk patients demonstrated worse treatment responses to ICIs and OS. This suggested that the scoring of the signature constructed herein could be used to predict the treatment responses to ICIs and OS. Although in our research we found that the expression of many m6A regulators was weakly correlated with PD-L1 expression, the signature of the m6A regulator related genes has no obvious correlation with PD-L1 expression. In immunotherapy, immune cell infiltration was the key to the effectiveness of ICI, and the expression level of PD-L1 was not an effective biomarker for ICI. The constructed signature can predict the efficacy of ICIs may be attributed to the fact that the signature was related to immune cells rather than PD-L1 expression.

Additionally, the high- and low-risk groups were found to present with different genetic mutation characteristics. The frequency of gene mutations in the high-risk group was higher than that in the low-risk group. A weak positive correlation was also observed between risk score and mutation load. Furthermore, the risk score was also weakly correlated with the tumor stemness index, immune score, and stroma score. These relationships might partly explain the underlying reasons as to why the risk scoring model constructed could be used to predict the prognosis of HNSCC.

In summary, this study has presented the following contributions to HNSCC research. First, it established a prognostic signature based on m6A regulator-related genes and validated its applicability *via* several methods. This signature can be used to effectively evaluate the prognoses of HNSCC patients and may also potentially predict responses to ICI treatments. Second, a few genes within this signature may be involved in the progression of HNSCC and may serve as potential therapeutic targets. Certain shortcomings and deficiencies in this study remain. First, this research was based on bioinformatics analysis, and the accuracy of the signature was not verified using clinical samples. Moreover, the role and regulation mechanism of these m6A regulator-related genes in HNSCC are still unclear, and follow-up research will further analyze the biological functions of these key genes in the occurrence and development of HNSCC.

## Conclusions

Our findings suggest that the developed prognostic signature, based on m6A regulator-related genes, can be used to effectively distinguish between the prognoses of HNSCC. This prognostic signature was shown to be related to the immune cell infiltration of HNSCC and might help predict the response and prognosis of ICI treatments. These findings suggested that the developed signature could be considered a broad-spectrum biomarker for prognosis in HNSCC and that it could be used to predict patients’ responses to ICIs.

## Data Availability Statement

The datasets presented in this study can be found in online repositories. The names of the repository/repositories and accession number(s) can be found in the article/**Supplementary Material**.

## Author Contributions

JC, TL, FZ, and XG designed the study. QL, MF, ZT, and YJ collected and analyzed data. JC and TL wrote the manuscript. JL and XG participated in the revision of the manuscript. All authors read and approved the manuscript.

## Funding

This work was supported by the National Natural Science Foundation of China (nos. 82160710, 82103478) and the National Cancer Center Climbing Fund (NCC201814B044, and NCC201814B040). This work was supported by the NHC Key Laboratory of Personalized Diagnosis and Treatment of Nasopharyngeal Carcinoma.

## Conflict of Interest

The authors declare that the research was conducted in the absence of any commercial or financial relationships that could be construed as a potential conflict of interest.

## Publisher’s Note

All claims expressed in this article are solely those of the authors and do not necessarily represent those of their affiliated organizations, or those of the publisher, the editors and the reviewers. Any product that may be evaluated in this article, or claim that may be made by its manufacturer, is not guaranteed or endorsed by the publisher.

## Supplementary Material

Supplementary Table 1 Data information used in this study.

Supplementary Table 2 1598 genes that are significantly correlated at all three levels.

Supplementary Table 3 65 enriched Kyoto Encyclopedia of Genes and Genomes signaling pathways.

Supplementary Table 4 187 genes in the top 10 enriched Kyoto Encyclopedia of Genes and Genomes pathways.

Supplementary Table 5 Top 10 enriched Kyoto Encyclopedia of Genes and Genomes pathways.

Supplementary Table 6 The correlation of m6A regulator expression and PD-L1 expression.

Supplementary Figure 1 Parts of genes that are highly correlated with m6A regulatory factors.

Supplementary Figure 2 Copy number variant and single nucleotide variant mutations of m6A regulator-related genes in key enriched pathways in head and neck, ovarian, cervical, endometrial, colorectal, and breast cancers.

Supplementary Figure 3 K-M survival curve of patients with high- and low-expression groups of certain key m6A regulator-related genes.

Supplementary Figure 4 Weak associations between tumor mutational burden (r = 0.11, *p* = 0.01), immune score (r = -0.15, *p* = 0.0008) and stromal score (r = 0.12, *p* = 0.01).

Supplementary Figure 5 The correlation of m6A regulator expression and PD-L1 expression. **(A)** YTHDC2, **(B)** YTHDF3 and **(C)** METTL14.

Supplementary Figure 6 The expression of PD-L1 between the two groups.
