# Supplementary figures and images for: A Signature of N6-methyladenosine Regulator-Related Genes Predicts Prognoses and Immune Responses for Head and Neck Squamous Cell Carcinoma
Source: Front Immunol. 2022 Feb 3;13:809872. doi: 10.3389/fimmu.2022.809872 (PMC8851317; doi:10.3389/fimmu.2022.809872)

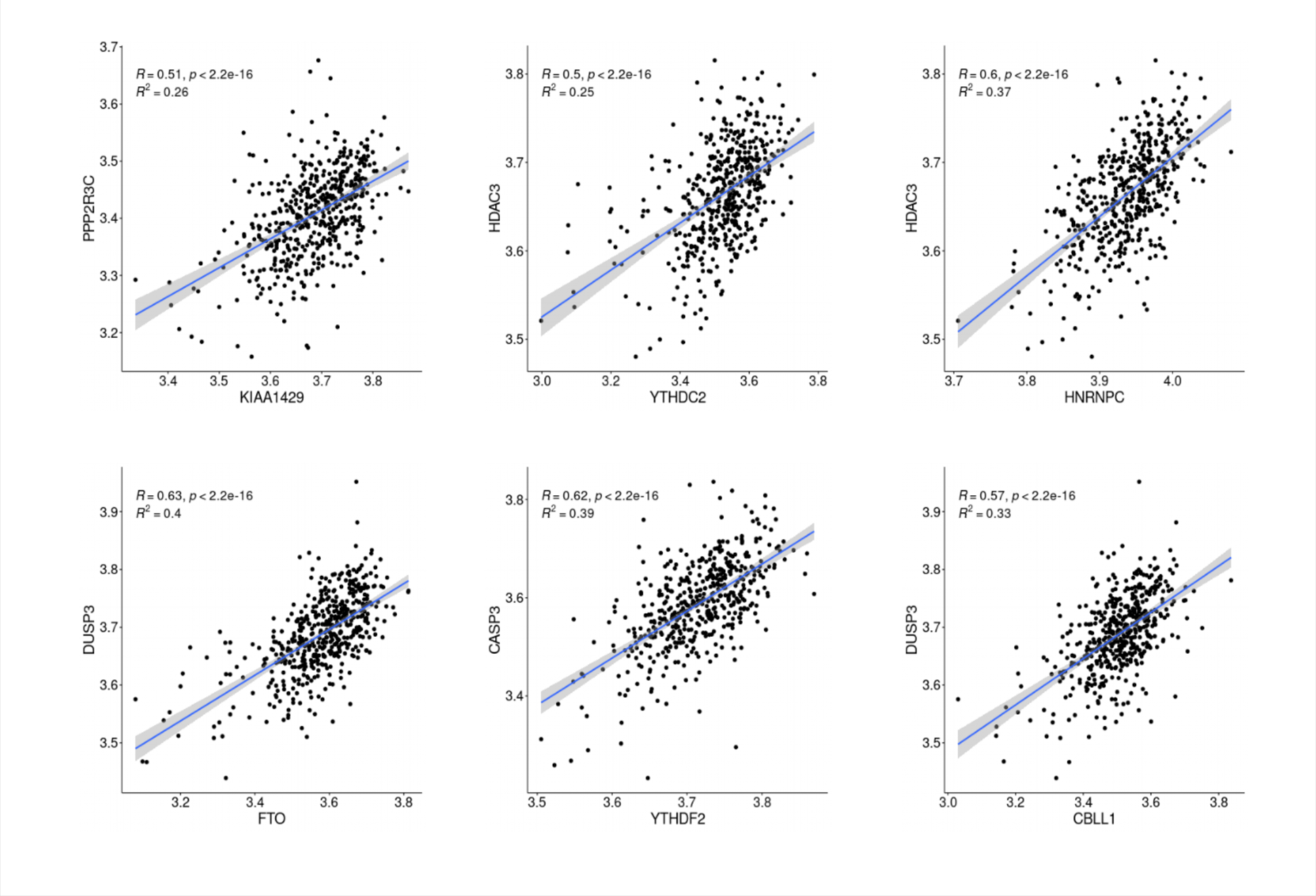

Supplement: Supplementary Figure 1 — Parts of genes that are highly correlated with m6A regulatory factors. [file Image_1.tif]

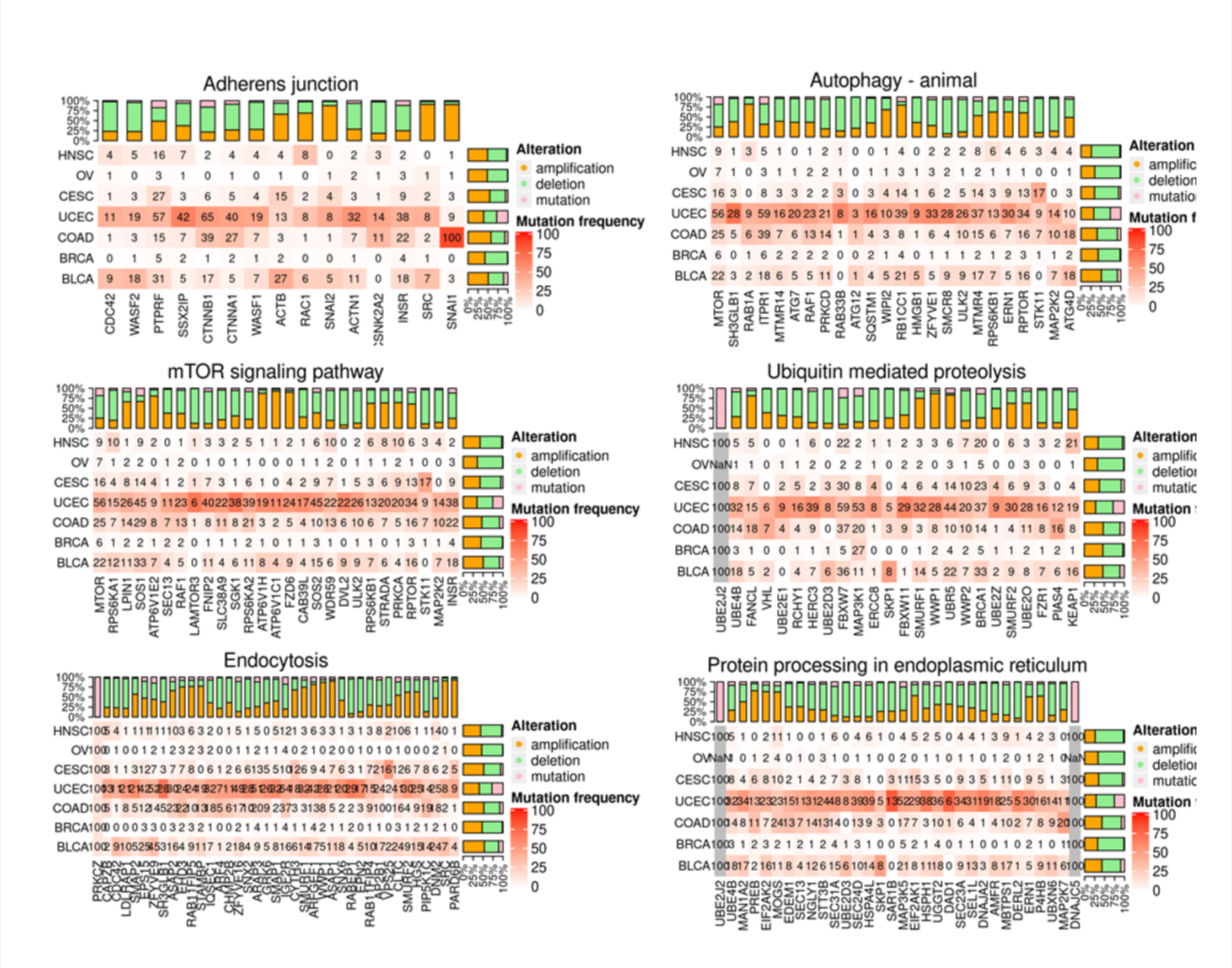

Supplement: Supplementary Figure 2 — Copy number variant and single nucleotide variant mutations of m6A regulator-related genes in key enriched pathways in head and neck, ovarian, cervical, endometrial, colorectal, and breast cancers. [file Image_2.tif]

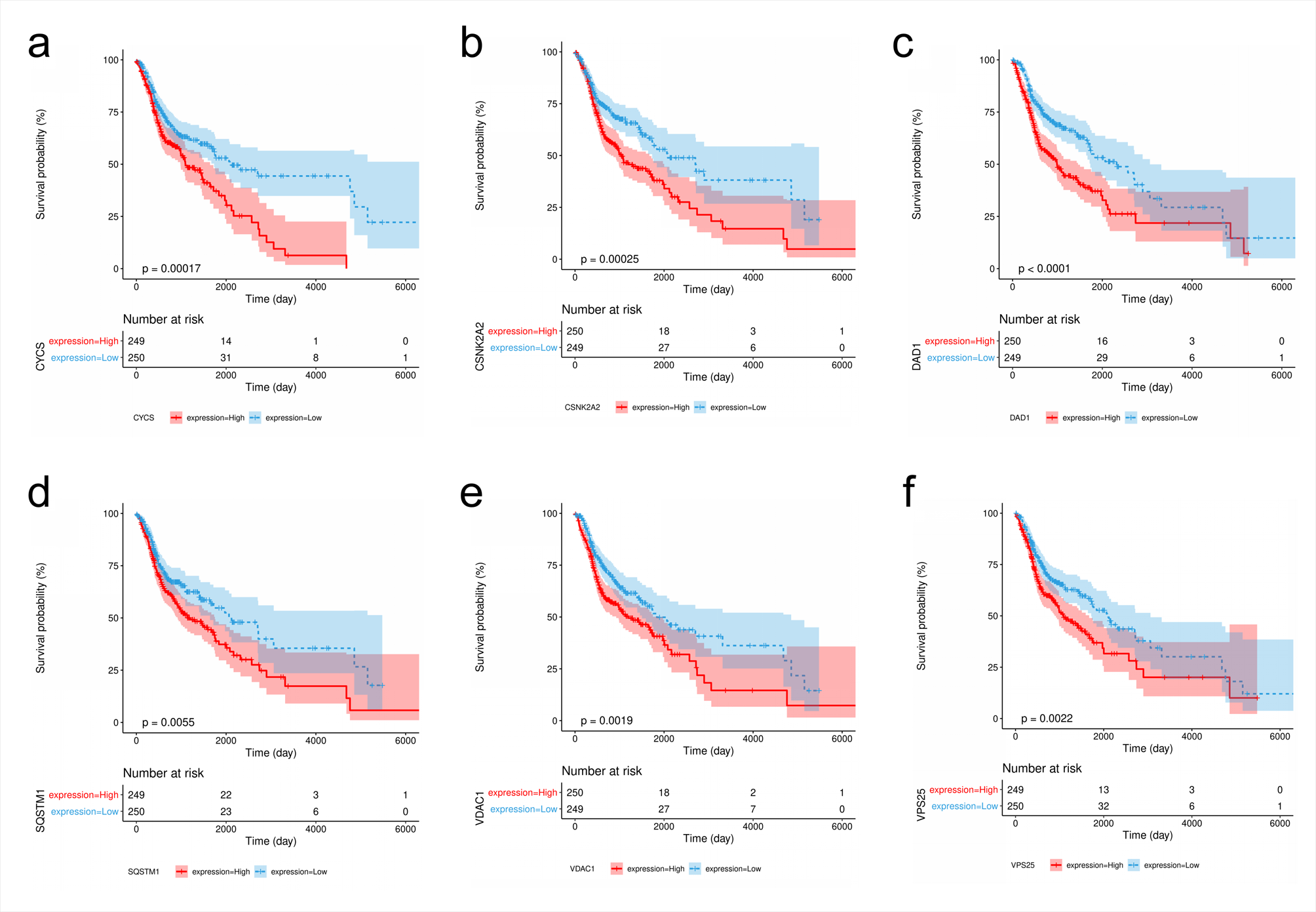

Supplement: Supplementary Figure 3 — K-M survival curve of patients with high- and low-expression groups of certain key m6A regulator-related genes. [file Image_3.tif]

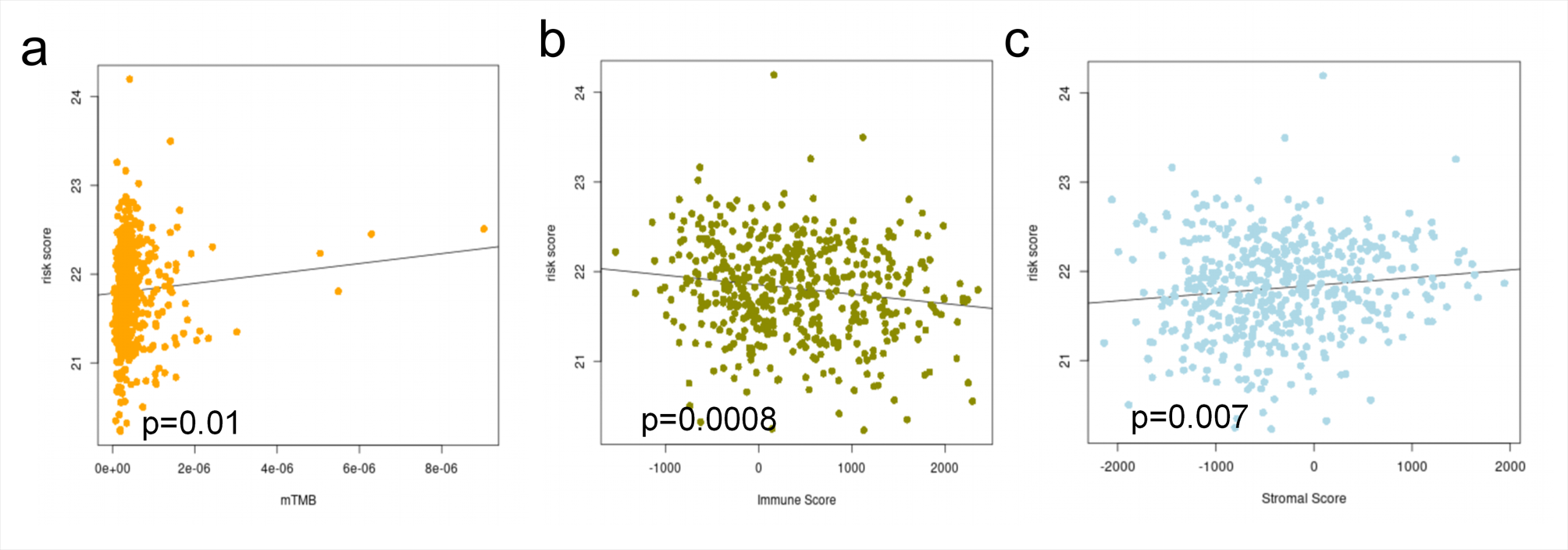

Supplement: Supplementary Figure 4 — Weak associations between tumor mutational burden (r = 0.11, p = 0.01), immune score (r = -0.15, p = 0.0008) and stromal score (r = 0.12, p = 0.01). [file Image_4.tif]

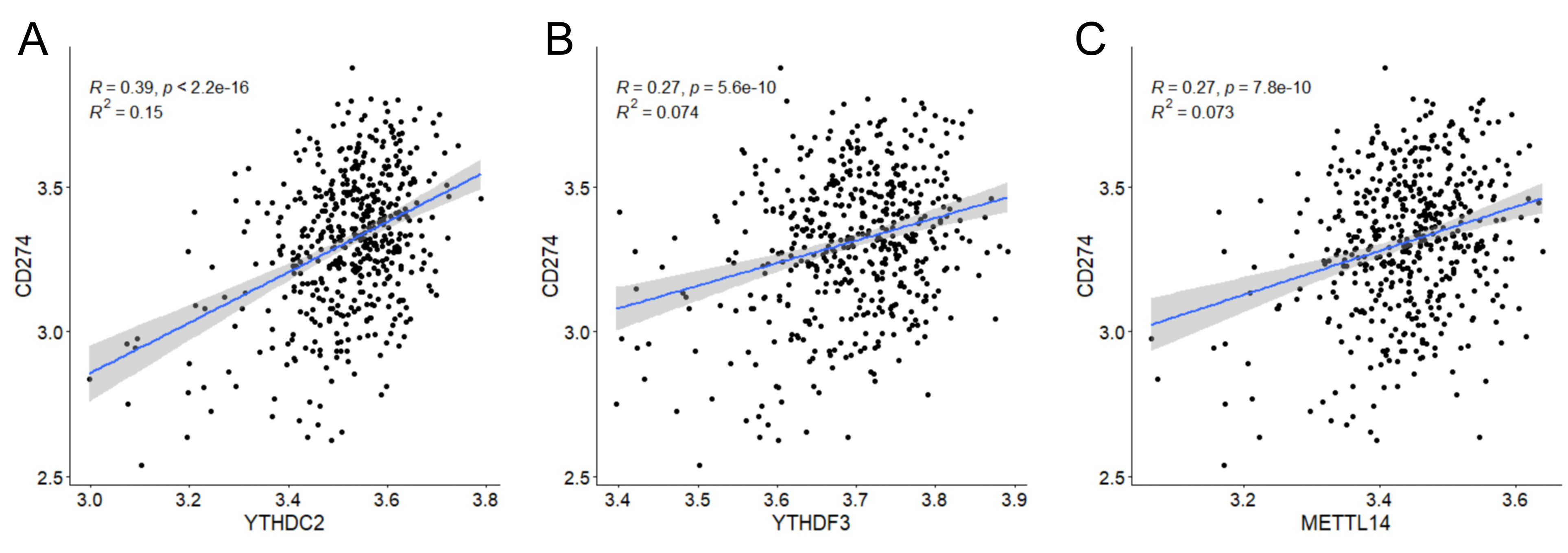

Supplement: Supplementary Figure 5 — The correlation of m6A regulator expression and PD-L1 expression. (A) YTHDC2, (B) YTHDF3 and (C) METTL14. [file Image_5.tif]

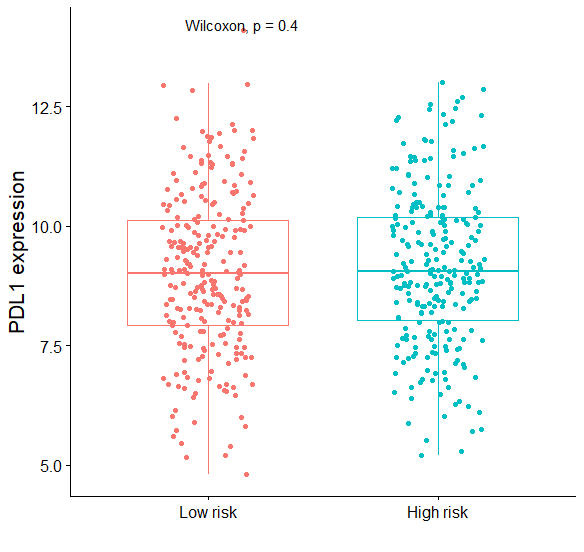

Supplement: Supplementary Figure 6 — The expression of PD-L1 between the two groups. [file Image_6.tiff]
